# Treinamento Resistido de Intensidade Moderada Melhora o Estresse Oxidativo no Coração

**DOI:** 10.36660/abc.20200561

**Published:** 2021-01-27

**Authors:** Marcelo Diarcadia Mariano Cezar, Silvio Assis de Oliveira, Ricardo Luiz Damatto

**Affiliations:** 1 Faculdade de Ciências Sociais e Agrárias de Itapeva ItapevaSP Brasil Faculdade de Ciências Sociais e Agrárias de Itapeva (FAIT), Itapeva, SP - Brasil; 2 Universidade Federal do Mato Grosso do Sul Campo GrandeMS Brasil Universidade Federal do Mato Grosso do Sul, Campo Grande, MS – Brasil

**Keywords:** Hipertensão, Estresse Oxidativo, Remodelação Ventricular, Treinamento de Resistência, Pressão Arterial/prevenção e controle

A hipertensão renovascular (HRV) é uma das principais causas de hipertensão secundária, muitas vezes levando à hipertensão resistente, ou seja, que não responde bem ao tratamento médico agressivo. Essa condição é definida como hipertensão arterial sistêmica que se manifesta em decorrência do comprometimento do suprimento sanguíneo para os rins.^1 , 2^ No contexto epidemiológico, a HRV é responsável por 1 a 5% de todos os casos de hipertensão e 5,4% da hipertensão secundária em adultos jovens.^3^

Estudos demonstram associação da doença com baixos níveis de atividade física ou aptidão física em hipertensos.^4 , 5^ Sabe-se que o treinamento físico tem ação protetora contra doenças cardiovasculares.^6 - 8^ Em 2016, a 7ª Diretriz Brasileira de Hipertensão Arterial, da Sociedade Brasileira de Cardiologia,^9^ reportou que a redução da pressão arterial é a medida mais eficaz para diminuir o risco cardiovascular e retardar a progressão dos danos renais, independentemente do anti-hipertensivo utilizado. O treinamento de resistência/exercício aeróbio promove importante efeito hipotensor em pacientes hipertensos e, portanto, tem sido recomendado como o tipo de exercício preferencial para prevenção e tratamento da hipertensão arterial.^9 , 10^

No entanto, é visto agora um interesse científico crescente nos efeitos cardiovasculares de outro tipo de exercício: o treinamento resistido.^11 , 12^ Treinamento resistido/força é uma atividade cujo esforço é realizado contra uma força oposta específica gerada pela resistência, projetada especificamente para aumentar a força e resistência muscular.^11^ Os efeitos benéficos do treinamento resistido abrangem a melhora do consumo máximo de oxigênio, da força e resistência muscular, além de ser um poderoso modulador do estresse oxidativo.^13^

Na edição atual da ABC, lemos com grande interesse o importante estudo entitulado “Treino de força reduz o estresse oxidativo cardíaco e renal em ratos com hipertensão renovascular”^14^ que aborda o impacto potencial de um protocolo de treinamento resistido sobre os danos oxidativos e nos sistemas enzimáticos antioxidantes endógenos no coração e rim contralateral em resposta à HRV. De fato, os animais com HRV induzida apresentaram características importantes da hipertensão, incluindo aumento da pressão arterial sistólica (PAS) e diastólica (PAD), pressão arterial média (PAM) e frequência cardíaca (FC). Animais sedentários hipertensos apresentavam elevada concentração de hidroperóxidos e níveis reduzidos de grupos sulfidrila.^14^

Os autores utilizaram um protocolo de treinamento resistido com 70% de sobrecarga de uma repetição máxima (1RM), com quatro séries de 12 repetições e intervalos de noventa segundos no decorrer de 12 semanas. Como consequência, os animais do grupo de hipertensos submetidos ao protocolo apresentaram redução nos valores de PAS, PAD, PAM e FC.^14^ É possível que o treinamento resistido tenha aumentado a disponibilidade de óxido nítrico e sua síntese pelas células endoteliais, contribuindo assim para a modulação do tônus vascular.^15^ Como consequência, o aumento da resposta bradicárdica poderia diminuir a atividade simpática no coração, levando a uma redução na FC de repouso, no débito cardíaco e nos níveis pressóricos.^4^

Outros achados foram: redução na concentração de hidroperóxidos e preservação de grupos sulfidrila no rim direito e no coração em animais hipertensos submetidos aos treinos. O grupo treinado apresentou aumento das atividades da superóxido dismutase (SOD), catalase e glutationa peroxidase no coração. Quanto ao rim em animais hipertensos, as atividades de SOD e catalase melhoraram em resposta ao treinamento resistido, embora a atividade da glutationa peroxidase não tenha se alterado.^14^ Exercícios regulares elevam a produção de espécies reativas de oxigênio (ERO) a um nível que pode induzir danos toleráveis, que, por sua vez, podem induzir adaptações benéficas ao regular positivamente os sistemas antioxidantes celulares e estimular os sistemas de reparo de danos oxidativos.^13^

Portanto, os resultados encontrados pelos autores indicam que o treinamento resistido de intensidade moderada pode ser uma intervenção eficaz no tratamento das doenças cardiometabólicas, principalmente a hipertensão renovascular. Porém, mais estudos são necessários para que possamos compreender os mecanismos moleculares relacionados ao balanço oxidativo em resposta aos treinos de força.
