# Supplementary material for: Treinamento Resistido de Intensidade Moderada Melhora o Estresse Oxidativo no Coração
Source: Arq Bras Cardiol. 2021 Jan 27;116(1):12–3. [Article in Portuguese] doi: 10.36660/abc.20200561 (PMC8159501; doi:10.36660/abc.20200561)
Supplement: Translation

# Translation: Moderate-Intensity Resistance Training Improves Oxidative Stress in Heart

**Authors:** Marcelo Diarcadia Mariano Cezar, Silvio Assis de Oliveira, Ricardo Luiz Damatto

**Affiliations:** 1 Brazil Faculdade de Ciências Sociais e Agrárias de Itapeva (FAIT), Itapeva, SP - Brazil; 2 Brazil Universidade Federal do Mato Grosso do Sul, Campo Grande, MS – Brazil

**Keywords:** Hypertension, Oxidative, Stress, Ventricular Remodeling, Resistance Training, Blood Pressure/prevention and control

Renovascular hypertension (RVHT) is one of the main causes of secondary hypertension, often leading to resistant hypertension, that is, that does not respond well to aggressive medical treatment. This condition is defined as systemic hypertension that manifests as a result of compromised blood supply to the kidneys.^1 , 2^In an epidemiological context, RVHT accounts for 1 to 5% of all cases of hypertension and 5.4% of secondary hypertension among young adults.^3^

Studies have shown an association this disease with low levels of physical activity or physical fitness in hypertensive individuals.^4 , 5^It is known that physical training has a protective action against cardiovascular diseases.^6 - 8^In 2016, the 7^th^Brazilian Guideline of Arterial Hypertension, by the Brazilian Society of Cardiology,^9^stated that blood pressure reduction is the most effective measure to decrease cardiovascular risk and slow kidney damage progression, regardless of the antihypertensive drug used. Endurance/aerobic exercise training promotes an important hypotensive effect in hypertensive patients and, therefore, has been recommended as the preferential type of exercise for arterial hypertension prevention and treatment.^9 , 10^

However, there is now a growing scientific interest in the cardiovascular effects of another type of exercise: the resistance exercise training.^11 , 12^Resistance/strength training is an activity whose effort is performed against a specific opposing force generated by resistance and which is designed specifically to increase muscular strength, resistance, and/or endurance.^11^The beneficial effects of resistance training encompass improved maximum oxygen consumption, muscle strength and endurance, in addition to being a powerful oxidative stress modulator.^13^

In the current edition of ABC, we read with great interest the important study “Strength Training Reduces Cardiac and Renal Oxidative Stress in Rats with Renovascular Hypertension”,^14^which addresses the potential impact of a resistance training protocol on oxidative damage and endogenous antioxidant enzymatic systems in the heart and contralateral kidney in response to RVHT. Indeed, the animals submitted to RVHT induction showed important characteristics of hypertension, including increase in systolic (SBP) and diastolic (DBP) blood pressure, mean blood pressure (MBP) and heart rate (HR). Sedentary hypertensive animals presented with an elevated concentration of hydroperoxides and reduced levels of sulfhydryl groups.^14^

The authors used a resistance training protocol with 70% overload of 1-repetition maximum (1RM), with four sets of 12 repetitions and ninety seconds intervals over a period of 12 weeks. As a result, the animals in the hypertensive group submitted to the resistance training protocol showed a reduction in the values of SBP, DBP, MBP, and HR.^14^Possibly, the resistance training has increased the availability of nitric oxide and its synthesis by endothelial cells, thus contributing to the modulation of vascular tone.^15^As a consequence, increased bradycardic response could decrease the sympathetic activity in the heart, leading to a reduction in HR at rest, in cardiac output, and in blood pressure levels.^4^

Other findings were reduction in the concentration of hydroperoxides and preservation of sulfhydryl groups in the right kidney and heart in trained hypertensive animals. The trained group presented enhanced superoxide dismutase (SOD), catalase and glutathione peroxidase activities in the heart. Regarding kidney in hypertensive animals, SOD and catalase activities were improved in response to resistance training, although glutathione peroxidase activity was unchanged.^14^Regular exercise elevates reactive oxygen species (ROS) production to a level that may induce tolerable damage, which in turn, can induce beneficial adaptations by upregulating cellular antioxidant systems and stimulating oxidative damage repair systems.^13^

Therefore, the results found by the authors indicate that resistance training of moderate intensity can be an effective intervention in the treatment of cardiometabolic diseases, especially renovascular hypertension. However, further studies are needed so we can understand the molecular mechanisms related to oxidative balance in response to resistance training.
